# Histopathological Classification of Breast Cancer Images Using a Multi-Scale Input and Multi-Feature Network

**DOI:** 10.3390/cancers12082031

**Published:** 2020-07-24

**Authors:** Taimoor Shakeel Sheikh, Yonghee Lee, Migyung Cho

**Affiliations:** 1 Department of Computer & Media Engineering, Tongmyong University, Busan 48520, Korea; sheikh@tu.ac.kr; 2 Department of Pathology, Ajou University School of Medicine, Ajou University Medical Center, Suwon 16499, Korea; yhl1227@aumc.ac.kr

**Keywords:** breast histopathology, computer-assisted diagnosis, whole slide imaging, multi-class classification, data augmentation

## Abstract

Diagnosis of pathologies using histopathological images can be time-consuming when many images with different magnification levels need to be analyzed. State-of-the-art computer vision and machine learning methods can help automate the diagnostic pathology workflow and thus reduce the analysis time. Automated systems can also be more efficient and accurate, and can increase the objectivity of diagnosis by reducing operator variability. We propose a multi-scale input and multi-feature network (MSI-MFNet) model, which can learn the overall structures and texture features of different scale tissues by fusing multi-resolution hierarchical feature maps from the network’s dense connectivity structure. The MSI-MFNet predicts the probability of a disease on the patch and image levels. We evaluated the performance of our proposed model on two public benchmark datasets. Furthermore, through ablation studies of the model, we found that multi-scale input and multi-feature maps play an important role in improving the performance of the model. Our proposed model outperformed the existing state-of-the-art models by demonstrating better accuracy, sensitivity, and specificity.

## 1. Introduction

Breast cancer has long been among the prevailing cancers in women [1,2,3]. With the development of digital imaging techniques, much attention has been devoted to the automatic detection and classification of cancers in whole-slide imaging (WSI). Current research in the field focuses on machine learning and deep neural network-based methods, directly impacting clinical and related studies, as well as the progression and development of targeted therapy approaches. Computational tools based on the digital slide workflow concept can help to increase efficiency and accuracy. Many methods have been developed for the analysis of pathological images, ranging from rule-based methods to machine learning-based ones [4]. Recently, deep learning-based approaches have been shown to outperform conventional machine learning methods, allowing automation of end-to-end processing [5,6,7]. In medical imaging, convolutional neural networks (CNNs) have been successfully used for diabetic retinopathy screening [8], bone disease prediction [9], age assessment [10], and other applications [5,11]. Previous deep learning-based approaches to the analysis of histological images have demonstrated their potential utility for breast cancer diagnostics [4,12,13,14] and for the micro-level analysis of pathological images [15,16].

In WSI, cancer cells can be observed at multiple levels (cellular and sub-cellular) and exhibit a variety of deviations from normal tissues. Variability of appearance in hematoxylin and eosin (H&E) stained areas is one of the major barriers to accurate image analysis [1]. These variations are caused by many factors, such as laboratory protocol variations, differences in the specimen orientation, operator-related variability, instrument variability, and use of different fluorophores for staining [15]. During diagnosis, detecting and categorizing these deviations can be difficult even for experienced pathologists. Pathologists use WSI at different magnification levels to make an accurate pathological diagnosis. Manual analysis of images is time-consuming and at times inaccurate; thus, an efficient and robust automated system is needed that will perform quick scanning and indicate potential pathological areas or disease-relevant regions of interest (ROIs). Such a computerized approach will not only increase efficiency, but will also enable more detailed and precise WSI analysis. The contributions of our research are as follows:

1. We proposed a multi-scale input and multi-feature network (MSI-MFNet) model based on a CNN, for the classification of breast cancer by training different scale image patches and learning not only the overall structures of cells but also their texture. Our proposed model fuses multi-resolution hierarchical feature maps with a dense connectivity structure in four different layers to extract more salient and diverse features.
2. We evaluated the performance of the MSI-MFNet model on two public benchmark datasets, showing that the proposed approach outperforms the existing state-of-the-art models, yielding the best accuracy, sensitivity and specificity results for various metrics (e.g., multiple magnification factors and binary and/or multi-class classification).
3. We verified how multi-scale input and multi-feature maps affect the performance of CNN-based models for cancer classification of histopathology images. To do this, we conducted ablation studies of our model, which applied a different number of multi-scale input and multi-feature maps to demonstrate the change in performance.

The remainder of this paper is organized as follows. In Section 2, we discuss related work. The architecture of the proposed MSI-MFNet model is presented in Section 3. In Section 4, we describe the datasets, experimental setup, model training, and implementation details. The detailed comparison of results are described in Section 5. Section 6 contains the discussion, conclusions, and potential future research directions.

## 2. Related Work

Significant efforts have been made to develop methods for breast cancer recognition and classification using histological images. Most existing methods focus on the classification of the two fundamental types of breast cancer (i.e., benign and malignant tumors) using computer-aided diagnosis (CAD) tools. Before the deep learning revolution, machine learning approaches, including support vector machine (SVM) [17], principle component analysis (PCA) [18], and random forest (RF) [19] methods were used to examine the data. The data features were extracted using scale-invariant feature extraction (SIFT) [20], local binary patterning (LBP) [21], completed LBP (CLBP) [22], local phase quantization (LPQ) [23], gray-level co-occurrence matrix (GLCM) [24], oriented FAST and rotated BRIEF (ORB) [25], and threshold adjacency statistics (PTAS) [26] approaches.

### 2.1. Traditional Learning Approaches

Most research in this area has been conducted using a very small number of samples from primarily private datasets. In 2013, a variety of algorithms, such as fuzzy C-means clustering, K-means clustering, competitive learning neural networks (NNs), and Gaussian mixture models were used for nuclei classification on a dataset with 500 real-case medical images collected from 50 patients. The accuracies reported for the binary classification task (benign versus malignant tumors) were in the 96–100% range [27]; thus, these machine learning-based methods enabled sufficiently accurate and objective analysis, and were deemed to be useful for facilitating breast cancer diagnosis. Another work was published by Spanhol et al., who reported 85.1% accuracy on a dataset of breast cancer histopathological images; that approach utilized an SVM and also used parameter free threshold adjacency statistics (PFTAS) features for patient-level analysis [28]. George et al. proposed a breast cancer recognition system based on NNs and an SVM, which reported 94% accuracy on a dataset consisting of 92 samples [29].

A cascading method with a rejection option was proposed by Zhang et al. This method was tested on a dataset of 361 samples from the Israel Institute of Technology. The reported accuracy was approximately 97% [30]. An effective breast cancer classification framework has been proposed with color texture features and multiple classifiers, such as SVMs, decision trees (DTs), nearest neighbor classifiers (NNCs), and discriminant analysis (DA). This method utilized ensemble voting with respect to the various classifiers, and the reported average patient-level recognition rate was 87.53% [31]. Different SVM-based techniques have been used for breast cancer recognition, with a reported accuracy of 94.97% for a dataset with a 40× magnification factor; this result was achieved using an adaptive sparse SVM (ASSVM) [32]. Several reviews have been published on histological image analysis for breast cancer detection and classification; these reviews clearly describe the dualities and limitations of different publicly available annotated datasets [1,33,34,35,36].

### 2.2. Deep Learning Approaches

Many breast cancer recognition methods have also used deep learning approaches. A few of these experiments were conducted using the famous BreakHis dataset. Bayramoglu proposed magnification-independent CNN and multi-task CNN models, where different-size convolution kernels (3 × 3, 5 × 5, and 7 × 7) were used; the study reported a patient-level accuracy of 83.25% [37]. Spanhol et al. proposed another model, similar to AlexNet, which uses various fusion techniques (i.e., sum, product, and maximum) for image- and patient-level analysis, and reported average accuracies of 90% and 85.6%, for the max fusion method applied to image and patient-level analysis, respectively [14]. Another deep learning-based study was published that, utilizes a pre-trained CNN model to extract feature vectors and then feeds the extracted vectors as an input to a classifier. This model, which was termed DeCAF, achieved accuracies of 86.3% and 84.2% for patient-level and image-level analyses, respectively [38]. Cruz-Roa et al. [39] proposed a CNN-based model for training 100 × 100 pixel patches for the detection of invasive carcinoma regions in breast histology slides. Their feature-extraction scale ranged from the nucleus level to the tissue level, and the method achieved an F1-score of 0.780.

A CNN-based model [12] was used to classify H&E stained breast biopsy images taken from another challenging dataset. For these images, multi-class classification (normal tissue, benign lesion, in-situ carcinoma, and invasive carcinoma) and binary classification (non-carcinoma as normal and benign tissue and carcinoma as in-situ and invasive carcinoma classes) were performed, and the results were evaluated on two levels: images and patches. A CNN-based approach achieved accuracies of 77.8% and 83.3% for multi-class and binary classification tasks, respectively, on the breast cancer Classification Challenge 2015 dataset [12]. Recently, Han et al. proposed a CSDCNN-based approach that was tested on the multi-class classification of breast cancer data from the BreakHis dataset and demonstrated state-of-the-art performance for both image- and patient-level classifications. An average accuracy of 93.2% for patient-level classification was reported [40]. Some studies have proposed the use of multi-scale input with deep learning approaches. For example, Cruz-Roa et al. [39] performed invasive carcinoma classification using a CNN model for 100 × 100 pixel patches, and each WSI was downsampled (by a factor of 16:1) to a resolution of 4 μm/pixel. Another method was proposed by Spanhol et al. [14] for classifying breast cancer images of different magnifications in benign and malignant tumors; the achieved accuracy was 84% for 200× magnification images.

## 3. Multi-Scale Input and Multi-Feature Network

**Figure 1:**
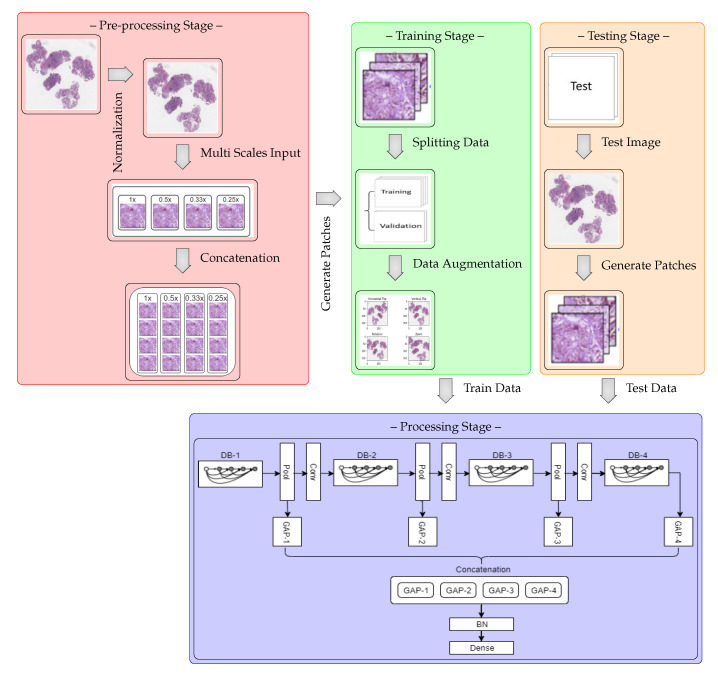
Block diagram of MSI-MFNet model. The blocks are DB: depth block (1–4); GAP: global average pooling (1–4); BN: batch normalization; Pool: pooling; and Conv: BN-ReLU-convolution.

The MSI-MFNet model is schematically shown in the block diagram in Figure 1, which shows that the model is trained on the training portion (train and validation sets) and then validated on the remaining test images. The model consists of four processing sub-blocks, which include different-depth blocks for exploring different-scale features, pooling and convolutional layers that represent the features on a range of scales, and global average pooling, which averages the output of each feature map before fusing the multi-feature map information, which prepares the model for the final classification layers (batch normalization and softmax layers).

The main reason for using multi-scale inputs from the normalized image is to learn the overall scale-variant structure of cells and their texture features at different levels simultaneously using different scales. This also overcomes the significant loss of resolution caused by the fixed receptive fields of the CNN and the pooling layers. Visual information also disappears from small regions during multiple convolutional and pooling operations, which results in poor performance with varied scales [41]. During the pre-processing stage, we used a simple H&E color normalization method [42] for the original raw image. To overcome the limitations associated with a spatial region comprising different scales, we re-scaled the normalized image to upscale ratios (1×, 0.5×, 0.33×, and 0.25×), which enhances the finer visual details of the important regions in the images. Then, each multi-scale input is divided into four patches prior to feeding it to the next network layer, as shown in the concatenation step.

A CNN is an organized hierarchically layered structure that, at each level, combines lower-level features into higher-level ones, until the desired image class label is obtained by minimizing the loss function [12,39,40]. The architecture of the MSI-MFNet is summarized in Table 1, with the following design considerations:

- **Input Layer (I):** This layer accepts the concatenation of resized patches (224 × 224 pixels) processed from the four different scale inputs (a,b,c,d), corresponding to re-scaled images from the original raw images with upscale ratios (1×, 0.5×, 0.33×, and 0.25×), respectively.
- **Depth Block (DB) and Feature Maps:** The tissues or ROIs vary in the target images; thus, to explore different-scale features, our network architecture has convolutional layers with a sufficient number of neural maps to represent each of these features on their range of scales, as shown in Table 1. The MF layer fuses the feature map information from four different learning layers for the entire image patch and performs the final classification.
- **Zero Padding (ZP):** This helps to preserve the original image size and provides useful information about feature learning, which helps to extract low-level features for each subsequent layer.
- **Max Pooling (MP):** Low-level information needs to be spatially integrated in the image region and with a more simplified representation when dealing with higher-level information. This layer deals with such complexity reduction without increasing the number of parameters in the network.
- **Batch Normalization (BN):** To increase the stability of a neural network, batch normalization normalizes the output of a previous activation layer by subtracting the batch mean and dividing by the batch standard deviation.
- **Average Pooling (AP):** We use this layer to perform down-sampling of the analyzed image by dividing the image into rectangular pooling regions and then computing the average values for each region to reduce the calculation complexity and parameters.
- **Non-Saturating (Non-linearity):** Both convolutional layers and fully connected layers are composed of rectified linear units (ReLUs). This helps manage the problem of vanishing gradients and also improves the training speed of the network [43,44].
- **Global Average Pooling (GAP):** This operation, applied to determine the average output of each feature map in the previous layer, reduces the data significantly and prepares the model for the final classification layers.
- **Output Layer (O):** The number of output neurons corresponding to each class, which are normalized using the softmax function; this number depends on the type of classification. In the present study, we performed binary and multi-class classifications (four or eight neurons).

## 4. Methodology

### 4.1. Datasets

We used the following two public datasets to validate our model. We chose these datasets for two reasons: the dataset size and the existence of multiple magnification factors, which allowed us to run several experiments on our restricted hardware by varying multiple criteria.

**Figure 2:**
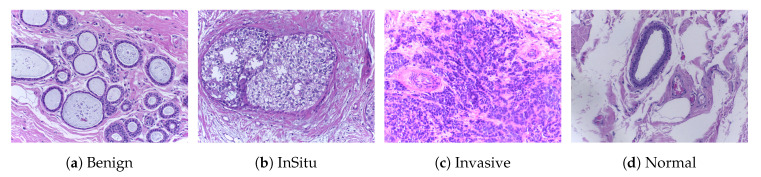
Microscopic H&E images of four types of tumors in the ICIAR2018 dataset. The magnification factor of these images is 200×.

***ICIAR2018*** [45]: This dataset (publicly available at https://iciar2018-challenge.grand-challenge.org/Dataset/) is an extended version of the Bioimaging 2015 breast histology classification challenge dataset and is described in [12]. The dataset consists of 400 H&E stained images (2048 × 1536 pixels) and includes four different classes. All of the images were digitized under the same acquisition conditions; the magnification is 200× and the pixel dimensions are 0.420 μm × 0.420 μm. As shown in Figure 2, each image is categorized into one of four classes: (1) benign, (2) in situ, (3) invasive carcinoma, and (4) normal; for each case, the assigned class corresponds to a predominant cancer type in the respective image. Image-wise annotation was performed by two medical experts. The goal of this challenge was to provide an automatic classification of each input image. In our experiments, we used a total of 492 images consisting of 400 samples for training and 92 samples for testing, and the annotation of these testing samples was performed by a pathologist. The structural details of this dataset are shown in Table 2.

**Figure 3:**
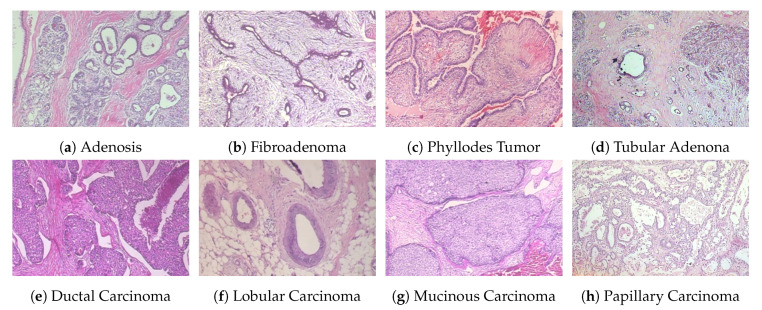
Four types of benign (first row) and malignant (second row) tumor images from the BreakHis dataset. The magnification factor of these images is 200×.

***BreakHis*** [14,28,38]: This dataset (publicly available at https://web.inf.ufpr.br/vri/databases/breast-cancer-histopathological-database-BreakHis/) contains a total of 7909 samples, each categorized as either benign or malignant. The subsets of benign and malignant samples contain 2480 and 5429 samples, respectively. The samples were collected from 82 patients with different magnification factors (40×, 100×, 200×, 400×), and the size of each image is 700 × 460 pixels. Each class has four subclasses, with the four types of benign tumors being adenosis, fibroadenoma, tubular adenoma, and phyllodes tumor. The four subclasses of cancer are ductal carcinoma, lobular carcinoma, mucinous carcinoma, and papillary carcinoma. The statistical details of this dataset are shown in Table 3, and some example histological images are shown in Figure 3. For our experiments, we used a random 70:30 partitioning of the entire dataset into training and testing subsets, respectively [1,40]. To evaluate the performance of our model for clinical situations, we ensured patient-based separation between training and testing data. That is, the images of a given patient were either all in the training set or all in the test set.

### 4.2. Image Representation

We used a simple H&E color normalization method [42] to normalize each image. We then resized the images into four different sizes with ratios of (1×, 0.5×, 0.33×, and 0.25×). These resized images are divided into four contiguous non-overlapping patches. After generating patches without augmentation, we concatenated them by resizing them into 224 × 224 pixels. We generated an augmented patch-wise dataset from the concatenated subset of four contiguous non-overlapping patches. In general, pathologists study histological images from various orientations, and there are many variations in staining and acquisition conditions. To mimic the pathologist examination process and realistic variation, we applied different types of data augmentation, such as flipping (horizontal and vertical), rotation, shifting (width and height), brightness, zoom, and blurring (slight). This data augmentation can increase the size of the dataset without deteriorating its quality. The literature also suggests that the data-augmentation and patching techniques can be used for histological classification [46]. Each of the generated patches was considered to have the same class label as the original image. These resized patches were used for training the model with image-net weights.

Image classification is performed by initially processing several different magnifications of patches using a patch-wise classifier, and then combining the results for the overall image patches to obtain a final image-wise classification. The classification of histological images into one of the target classes relies on the extraction and learning of related features, such as the overall tissue organization, the status of nuclei, and texture features. Nuclear features are useful for differentiating between carcinoma and non-carcinoma cells and include single-nucleus information, such as its shape, color, and structural features (e.g., density or variability). Conversely, information about the tissue structure is necessary to differentiate between in-situ and invasive carcinomas. Thus, the classification method uses the learned features on spatial scales ranging from that of the glands to the nuclear one. Visual inspection of the data suggests that the nuclear radius is in the 3- to 11-pixel range (1.260–4.62 μm). Based on this, we posited that 224 × 224 pixel patches should be sufficient to cover the relevant tissue structures of different cells.

The image-wise classification is performed using one of the following three patch probability fusion voting methods: (1) Majority voting: The image label is selected as the most occurring patch label; (2) Maximal probability voting: The patch with the highest class probability determines the image label; (3) Sum of probabilities voting: The patch class probabilities are summed and the class with the highest value is assigned. The final outcome was obtained by prioritizing malignant classes, using the following order: for the ICIAR2018 dataset (invasive, in-situ, benign, and normal) and for the BreakHis dataset (ductal carcinoma, lobular carcinoma, mucinous carcinoma, papillary carcinoma, adenosis, fibroadenoma, tubular adenoma, and phyllodes tumor).

### 4.3. Model Training

We evaluated the performance of the proposed model with respect to two different aspects: (1) classification of samples (binary and multi-class) and (2) the effect of data augmentation (with and without data augmentation). We used the BreakHis and ICIAR2018 datasets, as described in Section 4.1. The dataset was sub-divided into training (train and validation), and validation (test) subsets. We trained the model using 5-fold cross-validation on subset of training samples to determine the best hyper-parameters for model. During the testing phase, the model was evaluated only on the test portion of the dataset. We used accuracy, sensitivity, and specificity as the performance evaluation metrics. For these metrics, a higher score corresponds to a better-performing model on this classification task. To implement the method, we used the Keras framework on an NVIDIA Quadro RTX 5000. The metrics are reported after performing five successfully completed trials of experiments. We compared our model with the DNet [47] model, using the two different aspects mentioned above, as in the literature works, DNet yields the best classification performance.

### 4.4. Implementation Details

Our model comprises two main modules: (1) the feature extraction module, which learns the overall structures and texture features of microscopic-level tissues on different scales, and (2) the fusion of multi-resolution hierarchical feature maps from the dense connectivity structure. For feature learning, we used a CNN with a dense connectivity structure. The following seven hyper-parameters were tuned for the model: (1) the number of layers, (2) the number of epochs, (3) the learning rate, (4) the batch size, (5) the optimizer, (6) the dropout rate, and (7) batch normalization. An additional parameter that was common to all settings was the cross-entropy loss on the one-hot encoded output. We evaluated our model for layers 201, 205, and 210. The number of epochs assumed values of 30, 40, 50, and 70. The following learning rates were assessed: $10^{-1}$, $10^{-2}$, $10^{-3}$, $10^{-4}$, $5\times 10^{-1}$, and $5\times 10^{-2}$. For the batch size, we used 32, 64, and 128. We evaluated the following optimizers: SGD, RMSprop, Adadelta, Adam, Adamax, and Nadam. The dropout rates were 0.5, 0.6, 0.7, and 0.8. For the loss function, we used categorical cross entropy or binary cross-entropy because our input was in the [0,1] range. The best selected hyper-parameters with cross-validation technique are listed in Table 4.

## 5. Experiments and Results

### 5.1. Classification Results

Table 5 and Table 6 show the patch-wise classification results of binary and multi-class classifications for the two augmentation scenarios and for 200× magnification factors, for the two datasets. In terms of accuracy, our model outperformed the DNet model on the ICIAR2018 dataset. The accuracy increased to 83% for the without data augmentation scenario and 82% for the data augmentation scenario when only two classes were considered. Similarly for the BreakHis dataset, our model outperformed the DNet model by a large margin in the binary-class classification; the accuracy reached 90% and 98% for the without data augmentation and with data augmentation scenarios, respectively. However, clinically, multi-class classification results are more important for pathologists. Therefore, for both datasets, the accuracy results of our model were higher for both augmentation scenarios, which indicates that our model works well for this classification. The reason that the multi-class classification accuracy of the ICIAR2018 dataset is as low as 60% is because the distinction between normal and benign is ambiguous. A pathologist who labeled the test data said it was very difficult to distinguish between the two groups. In practice, pathologists often use immunity to accurately differentiate between normal and benign.

Our method demonstrated the highest sensitivity and specificity results for all cases of binary-class classification, compared with the multi-class classification task, while it demonstrated a stable classification performance regardless of the amount of variation (such as rotations and illumination) for the ICIAR2018 dataset. We observed that benign sensitivity is very low because pathologists have difficulty distinguishing benign from normal with only a few test images. For the BreakHis dataset, in terms of the sensitivity and specificity of binary classification, our model demonstrated better results than the DNet model. In addition, for multi-class classification, the sensitivity and specificity of our model outperformed that of the DNet model, except for a few cases that are comparable.

**Figure 4:**
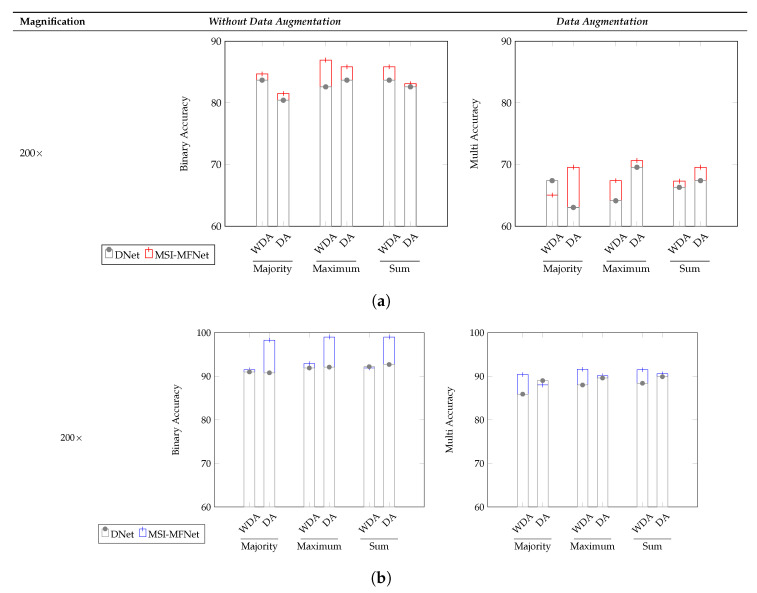
Image-wise comparisons of the accuracy metric on the (**a**) ICIAR2018 and (**b**) BreakHis datasets for the three different voting criteria (majority, maximum, and sum) and for the two augmentation scenarios WDA (without data augmentation) and DA (data augmentation).

The image-wise classification results for the three different voting criteria (majority, maximum, and sum) is shown in Figure 4 and in Table 7 and Table 8, denoting which are clinically more important and useful. For the ICIAR2018 dataset, the overall accuracy of our model for binary and multi-class classification increased, compared with that of the DNet model. As shown in Figure 4, the reason why the multi–class classification accuracy of ICIAR2018 data is significantly lower than the others is the same as that described before. Our model exhibited higher accuracy than the DNet model for all three voting criteria.

Table 7 lists the sensitivity and specificity results for the majority voting criterion for the ICIAR2018 dataset. In terms of both metrics, our model outperformed the DNet model with respect to both binary classification and multi-class classification. In the few cases where the model did not outperform DNet, this occurred because various biological structures overlapped in the respective WSI images; feature representations for such regions had no specific general structure, and these few regions varied in appearance compared with normal region variations. However, the sensitivity for the multi-class classification, except for benign classification, showed an average of over 90%.

The sensitivity and specificity results for the BreakHis dataset are reported in Table 8. Overall, our model outperformed the DNet model [47], except for a few cases. We assumed that this behavior for some cases was due to the scaling magnification images, which can sometimes affect structural information and make it more difficult to pick up relevant information from diseased regions; this is consistent with previous studies. Binary classification yields better results than multi-class classification for 200× and 400× magnification factors. We observed that the learned features are sensitive to variations in the data, which may be reflected in the changes in the selection of relevant regions. In contrast, the BreakHis dataset exhibits considerably large inter- and intra-class sample variations within each class, compared with the ICIAR2018 dataset. Class variations and their relationship to the number of samples in each class also affect the classification results.

For all of the considered criteria, the data augmentation scenario yielded a better performance than that without data augmentation, suggesting that it is a more suitable strategy for classification, as in general, medical images have several unique variants in which patches of images include different non-structural shapes, which can be further increased by augmentation of possible variations because of which general patterns can arise and assist the classification task. This, along with some amount of perspective blurring, improves the overall learning of relevant features. The best accuracy of patch-wise classification is explained by the fact that patch labels are obtained from image labels without any information about the location of abnormalities in medical images. Our approach is optimal as, regardless of the image class, normal tissue regions may be present. As a result, a small amount of noise (blurring) introduced in the training set does not, affect the patch-wise accuracy. Despite this, the network is able to focus on those details in the images that are relevant for classification. We also showed that the appearance variability of H&E stained regions can be improved, which is one of the major challenges in the analysis of breast cancer histopathological images [1].

### 5.2. Ablation Studies of MSI-MFNet

**Figure 5:**
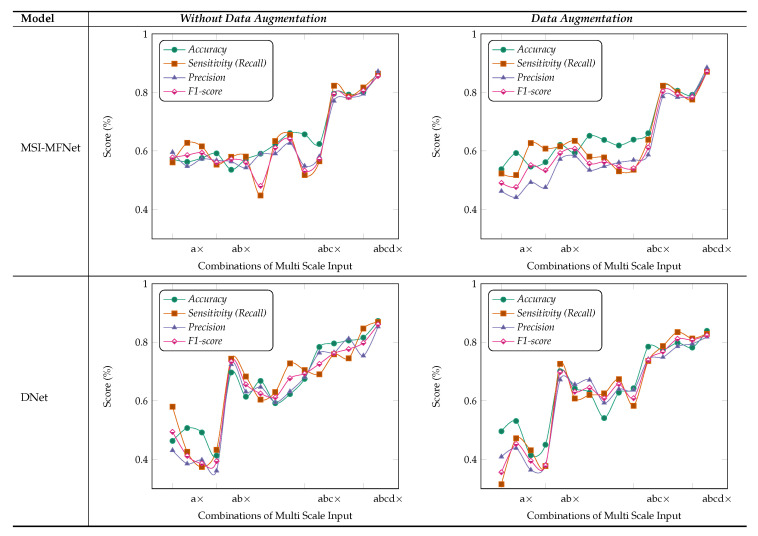
Results of ablation studies on the BreakHis dataset for MSI-MFNet and DNet with maximum voting criteria. Using the four different types of evaluation metrics, and we select a different combination of MSI fusion (i.e., 1×, 0.5×, 0.33×, and 0.25×) and a way of passing input data to MSI-MFNet, such as quadruplets, triplets, duplets, and individual raw features. For example: a× or ab× represents that we used only 1× or 1× and 0.5×, respectively.

**Figure 6:**
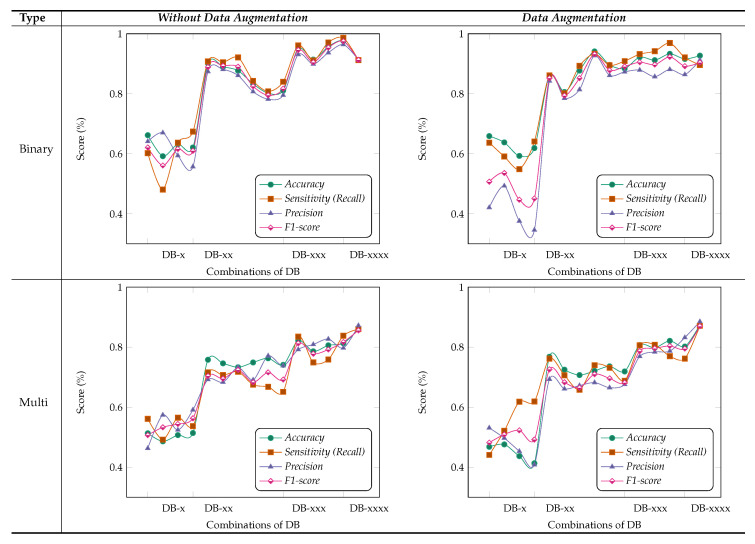
Results of ablation studies on the BreakHis dataset for binary and multi-class type classification with maximum voting criteria. Using the four different types of evaluation metrics, we select a different combination of MFNet maps and fuse them, i.e., DB-(1–4), such as quadruplets, triplets, pairs and individual concatenated feature maps. For example: DB-× or DB-××, represents that we used only DB-1 (first depth block) or DB-12 (first and second depth blocks), respectively.

We performed ablation studies using the MSI-MFNet model to gain deeper insights into the performance improvements associated with the different components of our model. The problem of multi-class classification has been thoroughly studied, with a special emphasis one why we used multi-scale input and multi-feature map modules in our model. Figure 5 and Figure 6 show the results of these studies, using four different metrics and varying different combinations (either single or multiple) of inputs and feature maps in each module. Experiments were performed using 400× magnification images from the BreakHis dataset.

***MSI (Multi-Scale Inputs) Module***: Figure 5 study shows that metrics can be improved using multi-scale images for classification of pathological images, where we considered different combinations of the MSI (i.e., 1×, 0.5×, 0.33×, and 0.25×), while employing fusion of all MFNet maps. For example, a× corresponds to using only 0.5× magnification images, while abc× corresponds to using 1× 0.5× and 0.33× magnification images. In the present work, we compared the performance of our model to the state-of-the-art DNet [47] model, because it yields the best performance in the literature. The results of our analysis show that training the models with multi-scale inputs has the benefit of learning the overall structural features and ambiguity regions. The proposed method obtained the highest metric results while maintaining a stable classification using the fusion of all multi-scale input combinations and data augmentation.

Moreover, results shown in Figure 5 demonstrate that our method learns representations that are robust to the scale of input, as demonstrated by the results when using multiple inputs. In contrast, for DNet experiments with data augmentation, there are variations in metric results, which are much lower with increasing number of multi-scale inputs as compared with the MSI-MFNet model. We also observed that our method without data augmentation does not outperform DNet (in a few cases) by selecting multi-scale input combinations that are triplets and duplets, which yields lower results according to all the metrics. This trend can be seen in MSI-MFNet and DNet model for without data augmentation scenarios. The accuracy and sensitivity metrics show comparable performance with increasing number of multi-scale inputs for MSI-MFNet as compared with the DNet model.

We believe that, in general, the multi-scale input behavior can improve the model performance because it allows control of the model results with different combinations of multi-scale inputs. By increasing MSI, we can increase the performance, so the model can accurately classify the cancer regions. Note that more combinations of multi-scale input introduce variations for model learning, by introducing diversity in data. Hence, by incorporating this module, the model learns a good decision capability and also demonstrates enhanced functionality to distinguish between the classes that can be too different from trained classes at prediction time, which are in general close enough to the realistic samples. The experimental setup with multi-class data shows the validity of our model utilizing this module, which improves the metrics with respect to the basic flat input approach of traditional CNN’s.

***MF (Multi-Feature Maps) Module***: Figure 6 shows the results obtained for different combinations of multi-feature maps in the DB-Depth Block DB-(1–4), where we considered using different numbers of depth blocks in the MSI-MFNet, while using a fusion of all MSI images (i.e., 1×, 0.5×, 0.33×, and 0.25×). For example, DB-× corresponds to using only one depth block (DB-1), while DB-xxx corresponds to using three depth blocks (DB-1, DB-2, and DB-3).

We observed that the model achieved the highest metric performance when we used a fusion of more combinations of multi-feature maps, which classify samples more accurately using rich information learned when data augmentation is applied to patches, compared with the scenario in which no data augmentation is employed, as shown in Figure 6. The data augmentation scenario of binary classification shows better performance compared with multi-class classification, with an overall average score of over 90%. We also noticed that there are several dips and changes in the metric scores for both augmentation scenarios and types of classification when fewer combinations of feature maps are used. We attribute this behavior to two factors: pair and individual feature maps learned are not useful for medical image classification because of large structural variations in images, and intra-class variations of samples also affect the model feature learning functionality.

Interestingly, we consistently obtained higher sensitivity scores for both types of classification with higher combinations of feature maps, which shows the capability of our model to correctly classify the patches with their respective classes. Nevertheless, our method still achieves a comparable performance with both scenarios of data augmentation when we used combinations of feature maps with more than pair combinations. Hence, in general, we can say that the classification models with more combinations of feature maps are more suitable and accurate for feature learning and to address multi-class task problems associated with medical images. However, from the experiments, we can state that the selection of multi-feature maps affects the results in various ways, but as already explained, in general, higher combinations show an improvement.

***Importance of MSI-MFNet***: Our model has two important characteristics. First, it has a powerful ability to transform raw data into high-level representation using different-scale image patches, which automatically bridges the semantic gap through image abstraction. This can be inferred by inspecting the results for two different models, as shown in Figure 5. The models’ performance is affected by input combinations; omitting one or more inputs from fusing reduces the models’ performance, that is, having more combinations of input scales is better. This trend was observed for all combinations for the both models. The results of our experiments demonstrate the need to fuse MSI for the classification of medical images. Second, Figure 6 shows the fusion results for different combinations of feature maps. These variations are reflected in the changes in the trends of different metrics, which also exhibit significant drops. These observations provide the rationale for fusing multi-resolution hierarchical feature maps at different layers. Such fusion allows the extraction of more distinct and salient information while retaining coarse-scale features. Fusing larger combinations of feature maps allows us to learn more robust and effective features for classification.

Fusing the feature maps from learning layers explicitly quantifies the fusion weights of the features used for classification, which is different from previous CNN-based methods [47,48,49] that typically fuse same-size feature maps and use encoding methods, which can corrupt the original spatial structure of the data features. A few studies have also proposed methods for designing parameters for generalization to arbitrary sizes, which means that information contained in different-size feature maps is not exploited to the fullest. We do not assume any input modalities that are explicitly fused or encoded using specific types of encoding operations. Instead, we rely only on raw multi-scale inputs, and later fuse feature maps from different layers. Unlike previous tightly coupled traditional methods [27,28,29], the present approach consists of separable modules: processing multi-scale inputs, feature learning with dense connectivity, fusing multi-scale feature maps, and classification layers. This modularity increases the method’s portability and applicability; consequently, the method can be used by other researchers for various medical image analysis tasks, such as visualization, classification, and segmentation.

Based on these ablation studies, we conclude that our results align with the objective of fusing multi-scale inputs before the feature learning stage and subsequent fusing of multiple feature map stages. Our general observation is that removing the fusion of combinations significantly affects the results, regardless of data augmentation, type of classification (binary or multi-class), or classification model, as shown in Figure 5 and Figure 6, respectively.

### 5.3. Confusion Matrix Visualization

**Figure 7:**
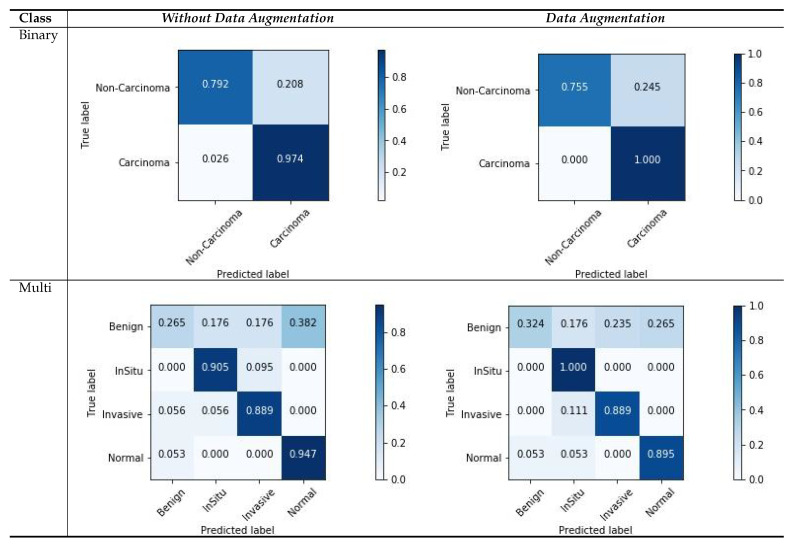
Confusion matrices for image-wise classification for the ICIAR2018 dataset, for the maximum voting criteria.

Figure 7 shows the confusion matrix results for the ICIAR2018 dataset with a magnification factor of 200×. From the visualization results, we can observe that the data augmentation scenario demonstrated better results than that without data augmentation. For both scenarios, there were several changes in the multi-class scores. This is due to the ambiguous distinction between normal and benign classes, as well as large intra-class sample variations within each class. The variations in the classes and their relationship to the number of samples in each class can also affect the classification results, and consequently, the image patches that are used for learning. However, the results of two classes of samples (In situ and Invasive) are more accurately classified. Moreover, our results demonstrate that the carcinoma classes were more precisely classified using binary classification with a data-augmentation scenario, as compared with non-carcinoma classes. This behavior reflects a smaller amount of data and weaker variations in the dataset. However, from these experiments, we can summarize that the use of data augmentation affects the results considerably, as demonstrated by the improved results.

**Figure 8:**
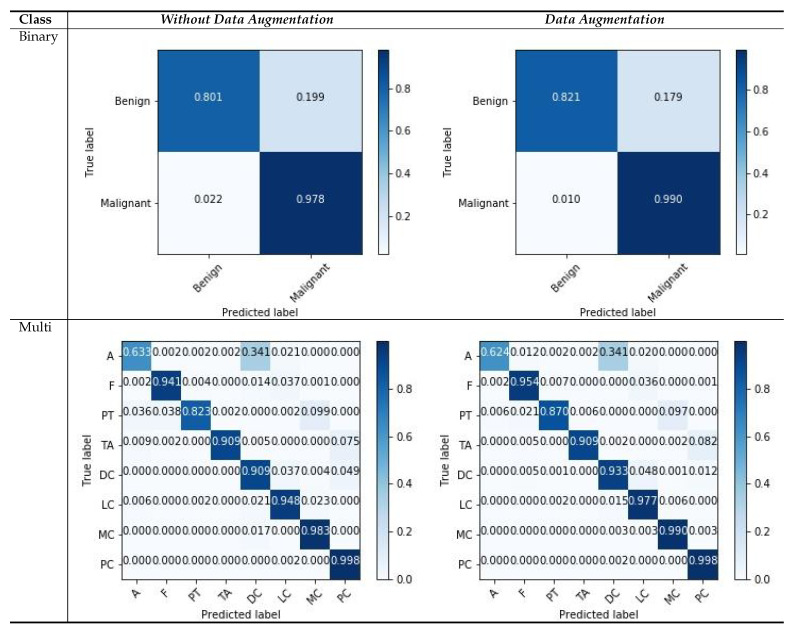
Confusion matrices for image-wise classification for the BreakHis dataset, for the maximum voting criteria.

Similarly, we show the confusion matrices of the BreakHis dataset with a magnification factor of 400× in Figure 8. We observe that our models tend to approximate good samples with deep layered networks, and input images with magnifications that are greater than 100× yield better results. From the visualization of the confusion matrix results, we can observe that the binary cases have superior performance compared with multi-classification cases, because of the rich and relevant regions, with data augmentation scenarios as compared to those without data augmentation. The features with higher magnification have good structural information, which helps the model to learn good representation between patches with respective labels. There are a few classes that consists of more patches without diseased regions and which deviate in appearance as compared with the normal region; such variations in patches can affect the classification capability, and the multi-classification results vary. Even with challenging variations in datasets, the visualization results suggest that data augmentation is a more suitable strategy for both types of classification. The overall result indicates that our model is useful for classifying histology images.

A factor that strongly improved the results was the application of data augmentation to patches, rather than the complete images, which sometimes exhibited no general patterns for feature learning. Carefully selecting each patch individually from WSI images and then applying data augmentation can further improve the model’s performance. However, such results are not useful for multi-scale networks and deviates from our objective of feature learning and the consequent fusion of feature maps from random patches. The overall results reported for both datasets and for the data augmentation scenarios suggest that our model performs well, regardless of the number of generated patches from WSIs, and using the fusion of combinations at the two modules of the MSI-MFNet model.

### 5.4. McNemar’s Statistical Analysis

Table 9 show the standardized McNemar’s test [50], which is employed to demonstrate the statistical significance in metrices improvement of the proposed MSI-MFNet. We design the comparison between MSI-MFNet and DNet model for all kinds of experimental results. The numbers for each dataset are computed using the comparison of ground truth labels with predicted labels of their respective model to form the McNemar table. Then for each dataset, we added the resultant cells from cases to form the final reported table. We observed from the *p*-value of the ICIAR2018 dataset, that our model didn’t outperform with large margin to DNet but still achieves comparable performance, it is because of the dataset have considerably fewer test samples for each class. For the BreakHis dataset, *p*-value is 0.08 which shows that the two models are statistically different with a confidence level of 92%, the statistical capability proves that our model have better classification capability, to address multi-class task problems associated with medical images. From these experiments, we can state that the proposed MSI-MFNet can significantly outperform literature method.

## 6. Conclusions

We proposed an MSI-MFNet model for classification of histological images by training different-scale image patches to learn the overall structures and texture features of cells using fusion of multi-resolution hierarchical feature maps at different layers. Our model yields classification probabilities on the patch and image levels. The model performance and strength were evaluated on two publicly available benchmark datasets, and for a variety of experimental strategies, such as multiple magnification factors, binary or multi-class classification, and either with or without data augmentation. Our model outperformed existing state-of-the-art models with respect to both datasets. We also performed an ablation study to gain deeper insights into the performance associated with the different components of our model, which verified the importance of multi-scale input and multi-feature maps. The proposed model achieved good sensitivity and specificity for different cases, which is useful for pathologists and researchers working in the field of cancer diagnosis using histological images. In future studies, we intend to investigate the model’s performance on other datasets that provide diverse cancer cases. We will also investigate which combinations of feature maps are likely to be most important for classification. The proposed system can be adapted for diverse tasks associated with histological image-based classification with relevance to clinical settings. 

## Figures and Tables

**Table 1:** MSI-MFNet architecture. Note that each “Conv” layer shown in the table corresponds to the sequence BN-ReLU-convolution. The blocks are as follows: MSI: multi scale input; I: input; DB-x: depth block (1–4), MF: multi feature and O: output.

Block	Layers	Output Size H × W	Parameters	Repetition	# of Param.	% of Param.
MSI	Input-1	a × a	Upscale 1x	-	-	-
	Input-2	b × b	Upscale 0.5x	-	-	-
	Input-3	c × c	Upscale 0.33x	-	-	-
	Input-4	d × d	Upscale 0.25x	-	-	-
I	Concat MSI (1–4)	224 × 224	-	-	-	-
	ZP	230 × 230	1 × 1	-	-	-
	Convolution	112 × 112	7 × 7, Stride 2	-	9408	0.1
	BN	112 × 112	-	-	256	-
	Activation	112 × 112	ReLU	-	-	-
	ZP	114 × 114	1 × 1	-	-	-
	MP	56 × 56	3 × 3, Stride 2	-	-	-
DB-1	Conv	56 × 56	1 × 1	× 6	338,304	1.8
	Conv		3 × 3		
	Conv	56 × 56	1 × 1	-	33,792	0.2
	AP	28 × 28	2 × 2, Stride 2	-	-	-
	GAP-1	128	-	-	-	-
DB-2	Conv	28 × 28	1 × 1	× 12	930,048	4.9
	Conv		3 × 3		
	Conv	28 × 28	1 × 1	-	133,120	0.7
	AP	14 × 14	2 × 2, Stride 2	-	-	-
	GAP-2	256	-	-	-	-
DB-3	Conv	14 × 14	1 × 1	× 48	8,180,736	43.3
	Conv		3 × 3		
	Conv	14 × 14	1 × 1	-	1,612,800	8.8
	AP	7 × 7	2 × 2, Stride 2	-	-	-
	GAP-3	896	-	-	-	-
DB-4	Conv	7 × 7	1 × 1	× 32	7,083,520	37.3
	Conv		3 × 3		
	GAP-4	1920	-	-	-	-
MF	Concat GAP (1–4)	3200	-	-	-	-
O	Dropout	3200	-	-	-	-
	BN	3200	-	-	12,800	0.1
	Softmax	2/4/8	-	-	25,608	0.1

**Table 2:** Structure of the ICIAR2018 with 200× magnification factor.

Classes	Subtypes	Magnification Factor (200×)		Total
		Training (Train & Validation)	Testing	
Non-Carcinoma	Benign (B)	100	35	135
	Normal (N)	100	18	118
Carcinoma	In situ (IS)	100	21	121
	Invasive (IV)	100	18	118
Total		400	92	492

**Table 3:** Structure of the BreakHis dataset with four magnifications (40×, 100×, 200×, and 400×).

Classes	Subtypes	Magnifcation Factors				Total	# of Patients
		40×	100×	200×	400×		
Benign	Adenosis (A)	114	113	111	106	444	4
	Fibroadenoma (F)	253	260	264	237	1014	10
	Tubular Adenona (TA)	109	121	108	115	453	3
	Phyllodes Tumor (PT)	149	150	140	130	569	7
Malignant	Ductal Carcinoma (DC)	864	903	896	788	3451	38
	Lobular Carcinoma (LC)	156	170	163	137	626	5
	Mucinous Carcinoma (MC)	205	222	196	169	792	9
	Papillary Carcinoma (PC)	145	142	135	138	560	6
Total		1995	2081	2013	1820	7909	82

**Table 4:** Best hyper-parameters of the MSI-MFNet and DNet classification models.

DataSet	Parameters	*Without Data Augmentation*		*Data Augmentation*	
		MSI-MFNet	DNet	MSI-MFNet	DNet
ICIAR2018	Number of layers	205	201	205	201
	Epochs	32	32	64	64
	Learning Rate	$10^{-3}$	$10^{-2}$	$10^{-4}$	$10^{-3}$
	Batch Size	Adam	Adam	Adam	Adam
	Dropout Rate	0.5	0.6	0.5	0.6
BreakHis	Number of layers	205	201	205	201
	Epochs	32	64	64	32
	Learning Rate	$10^{-4}$	$10^{-3}$	$10^{-4}$	$10^{-3}$
	Batch Size	Adam	Adam	Adam	Adam
	Dropout Rate	0.5	0.5	0.5	0.6

**Table 5:** Patch-wise comparisons of the accuracy, sensitivity, and specificity metrics for the ICIAR2018 dataset. The best results are shown in bold.

Class	Model	Accuracy		Sensitivity							
				***Without Data***				***Data***			
				***Augmentation***				***Augmentation***			
				***Non-Carcinoma***		***Carcinoma***		***Non-Carcinoma***		***Carcinoma***	
Binary	DNet	0.8233	0.8043	0.76		0.87		0.68		0.94	
	MSI-MFNet	**0.83**	**0.82**	**0.78**		**0.89**		**0.72**		**0.96**	
				***Benign***	***Normal***	***In Situ***	***Invasive***	***Benign***	***Normal***	***In Situ***	***Invasive***
Multi	DNet	0.601	0.631	0.300	**0.83**	0.763	0.710	0.264	0.927	0.805	0.75
	MSI-MFNet	**0.64**	**0.68**	**0.31**	0.717	**0.90**	**0.83**	**0.39**	0.932	**0.82**	**0.82**
				**Specificity**							
				***Without Data***				***Data***			
				***Augmentation***				***Augmentation***			
				***Non-Carcinoma***		***Carcinoma***		***Non-Carcinoma***		***Carcinoma***	
Binary	DNet			0.87		0.78		0.94		0.68	
	MSI-MFNet			**0.89**		**0.79**		**0.96**		**0.72**	
				***Benign***	***Normal***	***In Situ***	***Invasive***	***Benign***	***Normal***	***In Situ***	***Invasive***
Multi	DNet			0.879	0.809	**0.94**	0.819	0.963	0.788	0.892	0.853
	MSI-MFNet			**0.95**	**0.88**	0.872	**0.83**	**0.97**	**0.86**	**0.90**	**0.86**

**Table 6:** Patch-wise comparisons of the accuracy, sensitivity, and specificity metrics for the BreakHis dataset. The magnification factor is 200×, and other abbreviations (A, F, TA, PT, DC, LC, MC, and PC) are listed in Table 3. The best results are shown in bold.

Class	Model	Accuracy		Sensitivity															
				***Without Data***								***Data***							
				***Augmentation***								***Augmentation***							
				***Benign***				***Malignant***				***Benign***				***Malignant***			
Binary	DNet	0.896	0.909	0.75				0.97				0.75				0.99			
	MSI-MFNet	**0.92**	**0.98**	**0.76**				**0.99**				**0.94**				**0.99**			
				*A*	*F*	*PT*	*TA*	*DC*	*LC*	*MC*	*PC*	*A*	*F*	*PT*	*TA*	*DC*	*LC*	*MC*	*PC*
Multi	DNet	0.838	0.861	0.601	0.843	0.718	0.837	0.862	**0.85**	0.97	0.909	0.611	**0.87**	0.673	0.776	**0.92**	0.893	0.945	0.936
	MSI-MFNet	**0.88**	**0.87**	0.595	**0.87**	**0.79**	**0.89**	**0.96**	0.745	**0.98**	**0.92**	**0.62**	0.861	**0.78**	**0.82**	0.890	**0.90**	**0.98**	**0.98**
				**Specificity**															
				***Without Data***								***Data***							
				***Augmentation***								***Augmentation***							
				***Benign***				***Malignant***				***Benign***				***Malignant***			
Binary	DNet			0.97				0.74				0.95				0.93			
	MSI-MFNet			**0.98**				**0.76**				**0.99**				**0.94**			
				*A*	*F*	*PT*	*TA*	*DC*	*LC*	*MC*	*PC*	*A*	*F*	*PT*	*TA*	*DC*	*LC*	*MC*	*PC*
Multi	DNet			0.979	0.986	0.973	0.988	0.878	0.962	0.972	0.971	0.982	0.975	0.973	0.979	0.904	0.936	0.951	0.963
	MSI-MFNet			**0.99**	**1.0**	**0.99**	**1.0**	**0.90**	**0.98**	**0.98**	**0.99**	**1.0**	**0.99**	**0.99**	**0.99**	**0.93**	**0.96**	**0.97**	**0.98**

**Table 7:** Image-wise comparisons of the sensitivity and specificity metrics with respect to the ICIAR2018 dataset for the maximum voting criterion. The best results are shown in bold.

Class	Model	Sensitivity (Maximum)							
		***Without Data***				***Data***			
		***Augmentation***				***Augmentation***			
		***Non-Carcinoma***		***Carcinoma***		***Non-Carcinoma***		***Carcinoma***	
Binary	DNet	0.77		0.89		**0.72**		1.0	
	MSI-MFNet	**0.79**		**0.97**		0.69		1.0	
		***Benign***	***Normal***	***In Situ***	***Invasive***	***Benign***	***Normal***	***In Situ***	***Invasive***
Multi	DNet	0.27	**1.0**	0.78	0.74	0.29	1.0	0.78	0.95
	MSI-MFNet	**0.29**	0.91	**0.88**	**0.95**	**0.32**	1.0	**0.83**	**0.96**
		**Specificity (Maximum)**							
		***Without Data***				***Data***			
		***Augmentation***				***Augmentation***			
		***Non-Carcinoma***		***Carcinoma***		***Non-Carcinoma***		***Carcinoma***	
Binary	DNet	0.89		0.77		1.0		0.70	
	MSI-MFNet	**0.97**		**0.80**		1.0		**0.72**	
		***Benign***	***Normal***	***In Situ***	***Invasive***	***Benign***	***Normal***	***In Situ***	***Invasive***
Multi	DNet	0.91	0.78	**0.95**	0.82	0.98	0.85	0.91	0.84
	MSI-MFNet	**0.97**	**0.90**	0.89	**0.86**	0.98	**0.89**	**0.93**	**0.85**

**Table 8:** Image-wise comparisons of the sensitivity and specificity metrics on the BreakHis dataset for the maximum voting criterion. The abbreviations (A, F, TA, PT, DC, LC, MC, and PC) are from Table 3. The best results are shown in bold.

Class	Magnification	Model	Sensitivity (Maximum)															
			***Without Data***								***Data***							
			***Augmentation***								***Augmentation***							
			***Benign***				***Malignant***				***Benign***				***Malignant***			
Binary	200×	DNet	0.74				0.98				0.77				0.98			
		MSI-MFNet	**0.76**				**0.99**				**0.97**				**0.99**			
	400×	DNet	**0.80**				0.92				0.82				0.98			
		MSI-MFNet	0.88				**0.98**				**0.84**				**0.99**			
			*A*	*F*	*PT*	*TA*	*DC*	*LC*	*MC*	*PC*	*A*	*F*	*PT*	*TA*	*DC*	*LC*	*MC*	*PC*
Multi	200×	DNet	0.61	0.90	0.78	0.87	0.89	**0.91**	0.98	0.96	0.61	0.89	0.72	0.83	**0.95**	0.91	0.97	0.98
		MSI-MFNet	**0.62**	**0.91**	**0.85**	**0.92**	**0.98**	0.76	**0.99**	**0.98**	**0.62**	**0.91**	**0.84**	**0.86**	0.93	**0.92**	**0.99**	**1.0**
	400×	DNet	0.59	0.94	0.82	0.89	0.90	0.66	0.90	0.93	0.61	0.89	0.58	0.85	0.96	0.83	0.90	0.97
		MSI-MFNet	**0.63**	**0.95**	**0.83**	**0.91**	**0.98**	**0.94**	**0.98**	**0.99**	**0.62**	**0.95**	**0.87**	**0.90**	**0.93**	**0.97**	**0.99**	**0.99**
			**Specificity (Maximum)**															
			***Without Data***								***Data***							
			***Augmentation***								***Augmentation***							
			***Benign***				***Malignant***				***Benign***				***Malignant***			
Binary	200×	DNet	0.74				0.98				0.76				0.98			
		MSI-MFNet	**0.76**				**0.99**				**0.96**				**0.99**			
	400×	DNet	0.80				0.96				0.81				0.98			
		MSI-MFNet	**0.87**				**0.98**				**0.82**				**0.99**			
			*A*	*F*	*PT*	*TA*	*DC*	*LC*	*MC*	*PC*	*A*	*F*	*PT*	*TA*	*DC*	*LC*	*MC*	*PC*
Multi	200×	DNet	0.60	0.90	0.80	0.86	0.91	**0.91**	0.98	0.97	**0.62**	0.90	0.74	0.84	0.93	0.93	0.96	0.99
		MSI-MFNet	**0.62**	**0.91**	**0.85**	**0.92**	**0.99**	0.78	**0.99**	**0.98**	0.61	**0.91**	**0.84**	**0.88**	**0.95**	**0.94**	**0.99**	**1.0**
	400×	DNet	0.60	0.94	0.82	0.90	**0.99**	0.66	0.91	0.92	0.62	0.91	0.57	0.86	0.92	0.86	0.92	0.98
		MSI-MFNet	**0.63**	**0.95**	**0.83**	**0.91**	0.91	**0.97**	**0.98**	**0.99**	**0.63**	**0.95**	**0.87**	**0.91**	**0.94**	**0.98**	**0.98**	**0.99**

**Table 9:** Statistical significance from the standardized McNemar’s test.

Parameters			*ICIAR2018*		*BreakHis*	
	***MSI-MFNet***		***DNet***			
			***Correct***	***Incorrect***	***Correct***	***Incorrect***
		***Correct***	254	14	773	89
		***InCorrect***	20	76	115	495
*p*-value			0.39		0.08	
